# Explaining socioeconomic inequalities in alcohol use disorder symptoms: the role of social capital and drinking motives

**DOI:** 10.1093/alcalc/agaf012

**Published:** 2025-04-08

**Authors:** Karen Schelleman-Offermans, Alessandro Sasso, Karlijn Massar, Cátia Pinto Teixeira

**Affiliations:** Department of Work and Social Psychology, Maastricht University, P.O. Box 616, 6200MD Maastricht, the Netherlands; Joint Research Centre (JRC), European Commission, Via Enrico Fermi 2749-21027, Ispra (VA), Italy; Department of Work and Social Psychology, Maastricht University, P.O. Box 616, 6200MD Maastricht, the Netherlands; Department of Work and Social Psychology, Maastricht University, P.O. Box 616, 6200MD Maastricht, the Netherlands

**Keywords:** social capital, alcohol use disorder, drinking motives, socio-economic health inequalities

## Abstract

**Introduction:**

Empirical evidence of the buffering effect of social capital and its underlying psychosocial mechanisms on socio-economic inequalities in alcohol use disorder (AUD) symptoms is limited. As socio-economic disadvantages often go together with deficits in resources and considering social capital’s beneficial effects on health, we hypothesized a stronger buffering (at high scores) and a cumulative disadvantaged effect (at low scores) of social capital on AUD symptoms among people reporting higher socio-economic disadvantage compared with their more advantaged counterparts. Additionally, we investigated whether this moderation effect was associated with drinking motives.

**Method:**

Three-hundred and sixty-five young adults participated in a cross-sectional online questionnaire measuring all model variables. First, we tested a moderation model, including AUD symptoms (DV), perceived socio-economic disadvantage (IV), and social capital (moderator). Secondly, we tested a moderated mediation model, additionally including drinking motives as mediators of the moderation effect tested in the first model.

**Results:**

In the case of high social capital, young adults reporting higher socio-economic disadvantage reported fewer AUD symptoms than their advantaged counterparts, which was associated with their lower endorsement of coping, enhancement, and social motives. When social capital was low, those reporting higher socio-economic disadvantages showed higher AUD symptoms than their advantaged counterparts, which was associated with their higher endorsement of coping motives only.

**Conclusion:**

Social capital can buffer (at high levels) or aggravate (at low levels) socio-economic inequalities in AUD symptoms, and drinking for coping, enhancement, and social motives may explain why this happens.

## Introduction

People from socio-economic disadvantaged backgrounds bear a disproportionately higher burden of negative alcohol-related consequences than their socio-economically advantaged counterparts, with heavy episodic drinking playing a significant role in these disparities (e.g. Loring 2014, Probst et al. 2020). This may suggest that alcohol is used as a coping mechanism by individuals facing stressors due to their socio-economic disadvantage (Heim et al. 2020, Schelleman-Offermans et al. 2022). On the other hand, (cognitive) social capital, which relates to the quality of social networks and encompasses perceptions of trust, reciprocity, and shared values within a neighborhood (Bourdieu 1986, Uphoff et al. 2013), may serve as a healthy coping mechanism protecting against the negative effects of socio-economic disadvantage (Bourguignon et al. 2020) as it strengthens support systems, including sense of belongingness and mutual respect (Brunner 1997). Social capital has indeed been shown to protect against negative health outcomes such as morbidity and mortality, risk of infectious diseases, and alcohol use disorder (AUD) (Weitzman 1978, Kawachi et al. 1997, Schultz et al. 2008, Bryden et al. 2013, Larm et al. 2016). Furthermore, social capital has been shown to mitigate the association between stressors related to perceived discrimination and increased substance use, suggesting that it may buffer for negative stress effects (Walsh et al. 2018).

In line with the “buffering hypothesis” (Uphoff et al. 2013, Choi and Jun 2022), high social capital may have a greater beneficial effect on health outcomes such as AUD symptoms for people experiencing socio-economic disadvantage than for those in more advantaged socio-economic positions, since it may compensate for the lack of resources people in a socio-economic disadvantaged position often experience (Schelleman-Offermans and Massar 2020, Massar et al. 2021, Schelleman-Offermans et al. 2024). Indeed, a systematic review of the relationship between social capital and socio-economic inequalities in health showed that, out of the 19 studies testing the buffering hypothesis, 11 confirmed its relevance (Uphoff et al. 2013). Additionally, the “cumulative disadvantage” hypothesis states that the effects of disadvantage in one context (e.g. socio-economic disadvantage) are amplified by “disadvantages” in other domains (e.g. low social capital) (DiPrete and Eirich 2006, Choi and Jun 2022). In other words, experiencing socio-economic disadvantage in combination with a low social capital is assumed to lead to stronger negative effects, compared with either people in more advantaged socio-economic positions reporting low social capital, or people reporting high socio-economic disadvantage and high social capital. These exacerbated effects on health outcomes for people experiencing socio-economic disadvantage when social capital is low might be due to social capital often being the only resource they can rely on.

Although the buffering and the cumulative disadvantage hypotheses are distinct, they do not contradict each other and can co-exist. If both hypotheses hold true, this indicates that social capital might be a particularly important leveraging factor for changing the socio-economic gradient in AUD symptoms as it can function “simultaneously” as a protective and a risk factor. This is particularly important for adolescents and young adults with a low socio-economic status since adolescence through young adulthood is a critical developmental period for tackling alcohol-related harm. It’s during this time that risky drinking behaviors often emerge, potentially interfering with the essential neurodevelopment and maturation processes occurring in the brain (Squeglia and Gray 2016, Hua et al. 2020). Nonetheless, very little empirical research has focused on the protective or risk enhancing effects of social capital on AUD symptoms in this developmental period. Furthermore, although socio-economic disadvantage seems to shape the endorsement of drinking motives (Schelleman-Offermans et al. 2022), no previous research has investigated how such buffering or aggravating effects of social capital on socio-economic gradients in AUD symptoms can be explained by drinking motives.

### The role of drinking motives in explaining cumulative disadvantage and buffering hypothesis

Research suggests that individuals facing socio-economic disadvantage may use alcohol as a coping mechanism (Heim et al. 2020, Schelleman-Offermans et al. 2022). Such a coping strategy is associated with drinking motives (Cooper 1994). According to the Motivational Model of Alcohol Use (Cooper 1994, Cox and Klinger 2004), four different drinking motives exist. Enhancement motives (positive, internal) are linked to drinking with the aim of enhancing pleasant emotional states, whereas coping motives (negative, internal) are associated with drinking to avoid stressful or unpleasant feelings. Conformity motives (negative, external) relate to drinking to avoid social rejection, whereas social motives (positive, external) are associated with drinking to enhance social gatherings (Cox and Klinger 2004). Coping and enhancement motives have consistently been shown to be associated with high alcohol use and related consequences, whereas social motives have been shown to be associated with high alcohol use only. On the other hand, conformity motives have shown inconclusive results regarding the association with alcohol use and related problems, mostly showing no or even negative associations (Schelleman-Offermans et al. 2011, Crutzen et al. 2012, Bresin and Mekawi 2021).

Few studies that investigated the link between socio-economic position and drinking motives support the mediating role of coping motives in explaining the effects of socio-economic disadvantage on alcohol outcomes. For example, a low individual socio-economic position (i.e. working-class occupations) has shown to be associated with a higher endorsement of coping motives and higher hazardous drinking rates (Heim et al. 2020). Also, coping motives are more endorsed among adolescents growing up in families with a low affluence (Schelleman-Offermans et al. 2022) and among people who live in deprived neighborhoods, where possibly stress levels are high and other healthier coping resources are scarce (Karriker-Jaffe et al. 2016, Martin et al. 2019). In addition to coping motives, enhancement motives may be used as a coping mechanism to deal with internal stressors by enhancing positive moods, creating a more even balance between negative and positive experiences (Cooper 1994, Cox and Klinger 2004). This indicates that a higher endorsement of coping and enhancement motives could potentially explain the possible stronger positive association between socio-economic disadvantage and AUD symptoms when social capital is low (cumulative disadvantage). On the other hand, the buffering hypothesis (high social capital serving as a healthy coping mechanism) may be explained by a lower endorsement of coping and enhancement motives among people experiencing socio-economic disadvantage and a high social capital. A lower endorsement of social motives may additionally explain the buffering hypothesis. This would especially be the case if a high social capital would lead to more opportunities for socializing in ways that do not (or cannot) involve alcohol for people experiencing high socio-economic disadvantage. A low/high social capital might increase/reduce fears of social rejection, which might be associated with higher/lower endorsement of conformity motives. Nevertheless, it is unlikely that conformity motives function as one of the explanatory pathways for the possible moderation effect of social capital on socio-economic inequalities in AUD symptoms, since often no, or even negative, associations are found between conformity motives and alcohol outcomes (Schelleman-Offermans et al. 2011, Crutzen et al. 2012, Bresin and Mekawi 2021).

### Current study

Despite a number of studies investigating the protective effect of social capital on alcohol-related disorders (Schultz et al. 2008), it is still not clear how associations between socio-economic disadvantage and AUD symptoms are moderated by social capital and what the underlying psycho-social mechanisms are. However, this is an important knowledge to effectively develop targeted interventions, since mechanisms might work differently for people in differing socio-economic positions and social contexts (Krieger 1994, Uphoff et al. 2013). Furthermore, it is especially important to investigate such mechanisms for adolescents and young adults, who are particularly at risk for developing negative alcohol-related consequences. It is during this developmental period where prevalence of hazardous alcohol use is high and where critical neurological maturation of brain structures occurs (e.g. Hua et al. 2020).

The aim of the current study is to test the buffering and accumulative disadvantage hypotheses of social capital regarding the association between socio-economic disadvantage and AUD symptoms and investigate whether drinking motives mediate this moderation effect. We hypothesize that high or low scores of social capital have the potential to decrease or increase the socio-economic gradient in AUD symptoms, respectively. We expect socio-economic disadvantage to be negatively associated with coping, enhancement, and social drinking motives, when social capital is high, which in turn will be associated with decreased AUD symptoms (buffering hypothesis). Additionally, a positive association between socio-economic disadvantage and coping and enhancement drinking is expected, in case social capital is low, which in turn, is expected to be associated with increased AUD symptoms (cumulative disadvantage hypothesis).

## Methods

### Sampling and procedure

The study was approved by Maastricht University’s Ethics Review Committee Psychology and Neuroscience (188_10_2_2018_S118) and informed consent was obtained. Convenience online sampling was used. Students of Maastricht University recruited participants (16–30 years) via their personal networks on social media (i.e. Facebook, Instagram, and LinkedIn), mainly from their country of origin (Germany, Croatia, Italy, and Hungary). An online questionnaire including CAPTCHA questions for attention checks was offered to participants in different languages (German, Hungarian, Italian, Croatian, and English) and forward–backward translation was performed. Participants were asked to fill out the survey with a duration of 10 min in their own languages (or in English) between May and June of 2022. Participation in the study was voluntary without any monetary incentive.

### Analytic sample

In the requested age range, 454 participants gave consent to participate in the study. We excluded 12 (2.6%) participants due to abstaining from alcohol since drinking motives can only be assessed among drinkers. Another 62 (13.7%) participants were excluded since they had quit the survey before answering questions about the included model variables or covariates, resulting in an analytic (case complete) sample of *n* = 365 (8.4%).

### Measures

#### Dependent variable

AUD symptoms were measured using the AUDIT-10 (Alcohol Use Disorders Identification Test), a 10-item measurement instrument (Cronbach’s α = .90) used to screen for harmful alcohol consumption (Babor et al. 2001) that has been largely validated (Reinert and Allen 2002). Examples of items “How often do you have six or more drinks on one occasion?” and “How often during the last year have you failed to do what was normally expected from you because of drinking?” [“never (0) to (nearly) daily (4)”]. Sum scores were included in the analyses with higher scores indicating more harmful alcohol use. A natural log transformation was used to adjust for non-normality.

#### Independent variable

We measured perceived socio-economic disadvantage with the MacArthur Scale of Subjective Social Status, a self-anchoring scale in the form of a pictorial social ladder, where individuals assess their standing in relation to others by considering income, education, and occupation (Adler et al. 2000). Participants were asked to self-assess where they see themselves on the ladder compared to others surrounding them ranging from “worst off; having the least money, low or no education and low or no income (1) to best-off; having money, higher education and the best jobs (10).” The MacArthur Scale has been proven to have sufficient construct as well as face validity (de Almeida et al. 2018) and have shown to be more strongly associated with well-being and health outcomes including stress levels, than objective socio-economic status (SES) measures like income and education, showing only weak correlation with such outcomes (Adler 2009, García et al. 2017).

Reversed coding of reported scores on the ladder was used in the analyses such that higher scores indicate a higher perceived socio-economic disadvantage. To be able to compare perceived socio-economic disadvantage between the countries included (as disadvantage can be perceived differently in different countries), a transformation into a continuous proportional rank score (ridit score) for each country separately was conducted. The ridit score indicates the proportion of respondents with scores on perceived socio-economic disadvantage ranging from 0 to 1, with the country sample means set at 0.5 (Bross 1958), and has been used in previous studies (Schelleman-Offermans et al. 2022, Chung et al. 2024). Higher values reflect higher perceived socio-economic disadvantage relative to the others within their country.

#### Moderator

Social capital was measured with four items (Cronbach’s α = .89) focusing on the cognitive dimension of social capital and derived from previously conducted studies (Barnes et al. 1997, Lochner et al. 1999, Stafford et al. 2008, Gariepy et al. 2013). Participants were asked to rate how they perceive their neighborhood with response options ranging from “Strongly Disagree” (1) to “Strongly Agree” (7). Items are “People in my neighborhood are willing to help their neighbors,” “People in my neighborhood share the same values,” “People in my neighborhood can be trusted,” and “Most people in my neighborhood are friendly.” Mean scores were used in the analyses, and higher scores indicate higher social capital.

#### Mediators

We used the Drinking Motives Questionnaire–Revised Short Form (Kuntsche and Kuntsche 2009) to measure the endorsement of enhancement (Cronbach’s α = .83), coping (Cronbach’s α = .95), social (Cronbach’s α = .92), and conformity (Cronbach’s α = .92) drinking motives in the last 12 months (three items per motive dimension). Items asked how often they drank alcohol in the last 12 months for example “to forget about your problems?” (coping); “because you liked the feeling?” (enhancement); “because it made social gatherings more fun” (social); “to fit in with a group you like” (conformity). A 7-point Likert scale ranging from “never” (1) to “always” (7) was used. Mean scores were used in the analyses and higher scores indicate a stronger endorsement. For the coping and conformity subscale, a natural log transformation was performed to adjust for non-normality.

#### Covariates

Age (in years) and sex (female, male, prefer not to say, other) were included as covariates in all analyses, because of the shown associations with drinking motives and/or AUD symptoms (e.g. White 2020, Schelleman-Offermans et al. 2022). No participants reported another sex than male or female and only one (*N* = 1) participant preferred not to report their sex. Sex was therefore recoded into “male” (coded into 1) and “female/prefer not to say” (coded into 0).

### Data analyses

**Figure 1:**
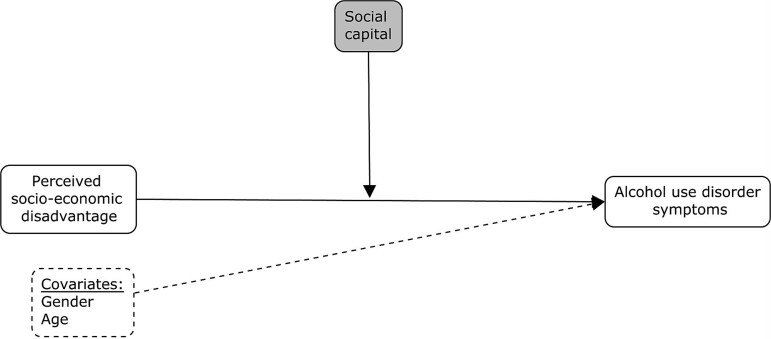
Conceptual analytic moderated model.

**Figure 2:**
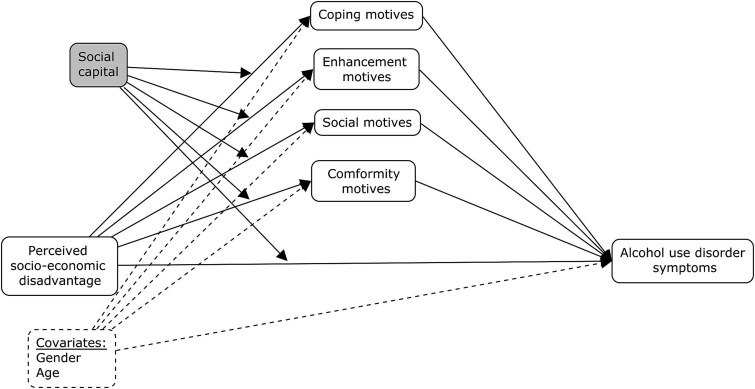
Conceptual analytic moderated mediation model.

Pearson or biserial correlations between model variables and multi-collinearity checks were conducted. The Hayes’ PROCESS Macro was used to test the proposed models. First, we tested a simple moderation model (Fig. 1) in which the interaction effect between perceived socio-economic disadvantage (independent variable) and social capital (moderator) on AUD symptoms (dependent variable) was tested. Secondly, a moderated mediation model was tested (Fig. 2), including 5000 bootstrap resampling, using AUD symptoms as dependent variable, perceived socio-economic disadvantage as an independent variable, all drinking motives as mediators and social capital as a moderator. All variables that define products in the tested models were mean-centered.

Bootstrap resampling cannot be combined with cluster analysis. To enable controlling for the clustered sampling design (participants clustered within countries/nationalities), a sensitivity analysis (using Mplus) was conducted to check the robustness of the significant findings resulting from the main moderated mediation model. We excluded nonsignificant pathways in these sensitivity analyses, to avoid that the number of parameters would exceed the number of clusters in the dataset. Furthermore, to ensure adolescents aged below 18 did not bias the results due to differences in legal alcohol availability with adult (18+) participants, additional analysis excluding participants below 18 (*n* = 17) was conducted.

**Table 1:** Descriptive statistics and Pearson and point-biserial correlations between model variables (*n* = 365).

	Mean (SD)/%	Range	1.	2.	3.	4.	5.	6.	7.	8.	9.
1. AUD symptoms^a^	7.657 (6.027)	1; 35	1								
2. Perceived socio-economic disadvantage	0.500 (.271)	0.001; .99	0.044	1							
3. Social capital	4.884 (1.222)	1; 7	−0.298^*^^*^	−0.191^*^^*^	1						
4. Coping^a^	2.469 (1.773)	1; 7	0.607^*^^*^	0.134^*^	−0.327^*^^*^	1					
5. Enhancement	3.722 (1.621)	1; 7	0.624^*^^*^	−0.020	−0.199^*^^*^	0.565^*^^*^	1				
6. Social	4.051 (1.693)	1; 7	0.590^*^^*^	−0.038	−.084	0.522^*^^*^	0.701^*^^*^	1			
7. Conformity^a^	1.074 (0.407)	1; 7	0.477^*^^*^	0.095	−0.170^*^^*^	0.506^*^^*^	0.429^*^^*^	0.487^*^^*^	1		
8. Age	24.21 (2.973)	17; 30	−0.100	−0.122^*^	0.085	0.008	−0.013	−0.033	−0.043	1	
9. Sex^b^ (male = 1; other = 0)	37.8	n.a.	0.265^*^^*^	−0.027	−0.155^*^^*^	0.061	0.109^*^	0.074	0.123^*^	−0.153^*^^*^	1

^**^ *P* < .01 (two-tailed); ^*^P < .05 (two-tailed).

^a^Pearson correlations are calculated using the log transformations;

^b^Point-biserial correlation and percentage reported.

## Results

### Descriptive results

Participants had a mean age of 24.09 (*SD* = 2.97; *M* = 25; range = 17–30; see Table 1 for descriptive results). The majority of participants reported to be female (61.08%). In total, participants from 32 different nationalities participated, including the Hungarian (*n* = 113; 31.00%), German (*n* = 97; 26.58%), Croatian (*n* = 65; 17.81%), Italian (*n* = 28; 7.67%), and Dutch (*n* = 13; 3.56%) nationality. The remaining number of participants (*n* = 49; 13.42%) reported other nationalities, mainly from different European countries, ranging from 1 to 5 participants per nationality.

No significant association was found between perceived socio-economic disadvantage and AUD symptoms. Coping and enhancement motives showed a significant strong positive and social and conformity motives a significant moderate positive correlation, whereas social capital showed a weak significant negative correlation with AUD symptoms. Perceived socio-economic disadvantage showed a weak significant positive association with coping motives, however, no associations with social, conformity, and enhancement motives. Social capital showed significant negative, but weak, correlations with perceived socio-economic disadvantage, coping, enhancement, and conformity motives, and no association with social motives.

### Results of moderation model

**Graph 1:**
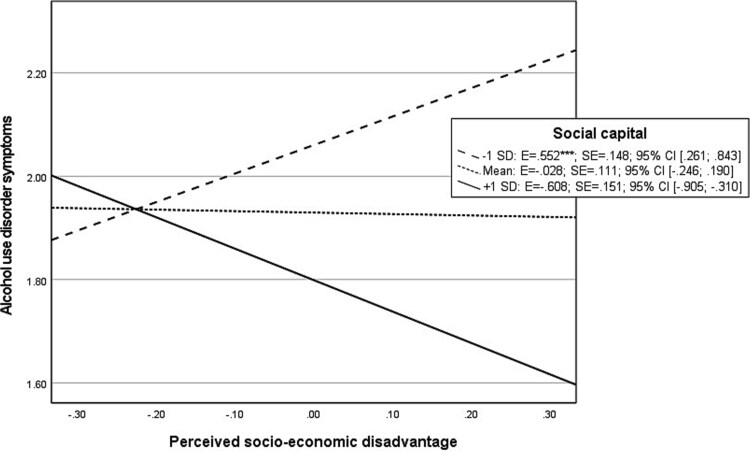
Probed interaction effect of perceived socio-economic disadvantage with social capital on natural log-transformed alcohol use disorder symptoms in moderation model. Footnote: ^*^P < .05; ^**^P < .01; ^***^P < .001.

The overall moderation model (see Table 2) showed to significantly explain 21.3% of the variance in AUD symptoms. A significant interaction was found between social capital and perceived socio-economic disadvantage on AUD symptoms. The probed interaction effects (Graph 1) display that perceived socio-economic disadvantage shows a significant positive association with AUD symptoms when social capital is low. Conversely, in case perceived social capital is high, perceived socio-economic disadvantage shows a negative association with AUD symptoms.

**Table 2:** Simple moderation analyses results (*n* = 365).

	DV = alcohol use disorder symptoms		
	*R* ^2^ = .213 *F*(5;359) = 19.434, *P* < .0001		
	*B* [95% CI]	*t* (SE)	
Perceived socio-economic disadvantage (PSED)	−0.028 [−0.246; .190]	−0.253 (.111)
Social capital (SC)	−0.107^*^^*^^*^ [−.157; −0.058]	−4.251 (0.025)
PSED^*^SC	−0.474^*^^*^^*^ [−0.636; −0.313]	−5.773 (0.082)
Age	−0.007 [−0.026; .013]	−0.660 (0.010)
Sex (male = 1; female/other = 0)	0.262^*^^*^^*^ [0.140; 0.383]	4.228 (0.062)

^***^ *P* < .001; ^**^P < .01; ^*^P < .05; SE, standard error.

### Results of moderation mediation model

The overall moderated mediation model (see Table 3) significantly explained 58.0% of the variance in AUD symptoms. Significant moderated mediation effects and conditional indirect effects were found for coping, enhancement, and social motives. Furthermore, after including motives as mediators in the model, the interaction effect between social capital and perceived socio-economic disadvantage decreased by >50%, indicating that this moderation effect was partly explained by these drinking motives. The significant moderated mediation indexes (Table 3) indicate that the significant negative effect found between socio-economic disadvantage and AUD symptoms at high levels of social capital (buffering hypothesis) is associated with the lower endorsement of coping, enhancement, and social drinking motives. On the other hand, the significant positive association found between socio-economic disadvantage and AUD symptoms at low levels of social capital (cumulative disadvantage hypothesis) is associated with a higher endorsement of coping motives only.

**Table 3:** Moderated mediation results (*n* = 365).

	DV = Coping		DV = Enhancement		DV = Social		DV = Conformity		DV = AUD symptoms	
	*R* ^2^ = .171 *F*(5;359) = 14.848, *P* < .0001		*R* ^2^ = .077 *F*(5;359) = 5.828, *P* < .0001		*R* ^2^ = .031 *F*(5;359) = 2.297, *P* < .045		*R* ^2^ = .088 *F*(5;359) = 6.951, *P* < .0001		*R* ^2^ = .580 *F*(9;355) = 54.460, *P* < .0001	
	*B* [95% CI]	t (SE)	B [95% CI]	t (SE)	B [95% CI]	t (SE)	B [95% CI]	t (SE)	B [95% CI]	t (SE)
PSED	0.126 [−0.036; 0.287]	1.530 (0.082)	−0.374 [−0.987; 0.239]	−1.200 (0.312)	−0.363 [−1.019; 0.293]	−1.088 (0.334)	0.103 [−0.050; 0.256]	1.330 (0.078)	−0.020 [−0.182; 0.143]	−0.237 (0.083)
SC	−0.098^***^ [−0.135; −0.062]	−5.273 (0.019)	−0.221^**^ [−0.360; −0.082]	−3.124 (0.071)	−0.081 [−0.230; 0.068]	−1.071 (0.076)	−0.034 [−0.068; 0.001]	−1.898 (0.018)	−0.043^*^ [−0.081; −0.005]	−2.228 (0.019)
PSED^*^SC	−0.301^***^ [−0.420; −0.181]	−4.952 (0.061)	−0.729^**^ [−1.183; −0.247]	−3.153 (0.231)	−0.619^*^ [−1.104; −0.133]	−2.504 (0.247)	−0.241^***^ [−0.355; −0.128]	−4.188 (0.058)	−0.228^**^ [−0.351; −0.104]	−3.618 (0.063)
Age	0.009 [−0.005; 0.024]	1.225 (0.007)	−0.007 [−0.063; 0.048]	−0.262 (0.028)	−0.010 [−0.070; 0.049]	−0.333 (0.186)	0.005 [−0.009; 0.019]	0.671 (0.007)	−0.009 [−0.023; 0.006]	−1.185 (0.007)
Sex	0.009 [−0.081; 0.099]	0.191 (0.046)	0.216 [−0.127; 0.558]	1.239 (0.174)	0.172 [−0.194; 0.538]	0.926 (0.186)	0.080 [−0.006; 0.165]	1.840 (0.043)	0.216^***^ [0.126; 0.306]	4.709 (0.046)
Coping									0.337^***^ [0.207; 0.468]	5.094 (0.066)
Enhancement									0.091^***^ [0.052; 0.130]	4.576 (0.020)
Social									0.081^***^ [0.043; 0.118]	4.230 (0.019)
Conformity									0.121 [−0.007; 0.249]	1.857 (0.065)
	**Index moderated** **mediation** **[Boot95% CI]**	**BootSE**	**Index moderated** **mediation** **[Boot95% CI]**	**BootSE**	**Index moderated** **mediation** **[Boot95% CI]**	**BootSE**	**Index moderated** **mediation** **[Boot95% CI]**	**BootSE**		
PSED	−0.102 [−0.162; −0.049]	0.029	−0.066 [−0.125; −0.016]	0.028	−0.050 [−0.104; −0.006]	0.025	−0.029 [−0.076; 0.007]	0.021		

PSE, perceived socio-economic disadvantage; SC, social capital; Boot, bootstrapped; SD, standard deviation; SE, standard error. In case 0 is included in the 95% CI, the effect is not significant.

**Graph 2:**
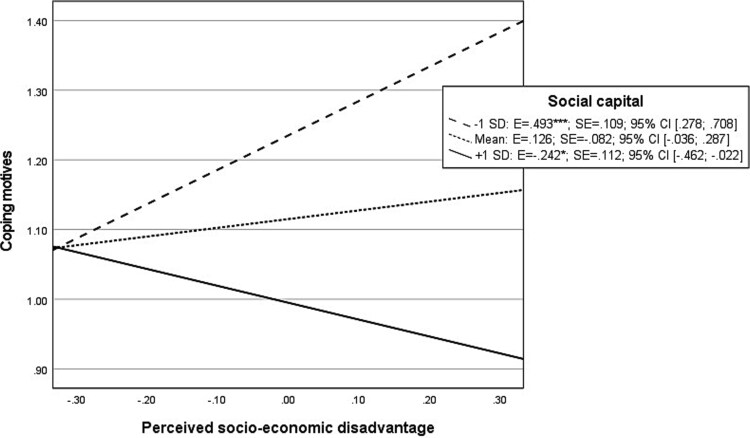
Probed interaction effect of perceived socio-economic disadvantage with social capital on natural log-transformed coping motives in moderated mediation analysis. Footnote: ^*^P < .05; ^**^P < .01; ^***^P < .001.

The probed interaction effect between perceived socio-economic disadvantage and social capital on coping motives (Graph 2) displays that at low levels of social capital, perceived socio-economic disadvantage showed a significant “positive” association with coping motives. In contrast, at high levels of social capital, perceived socio-economic disadvantage showed a significant “negative” association with coping motives.

**Graph 3:**
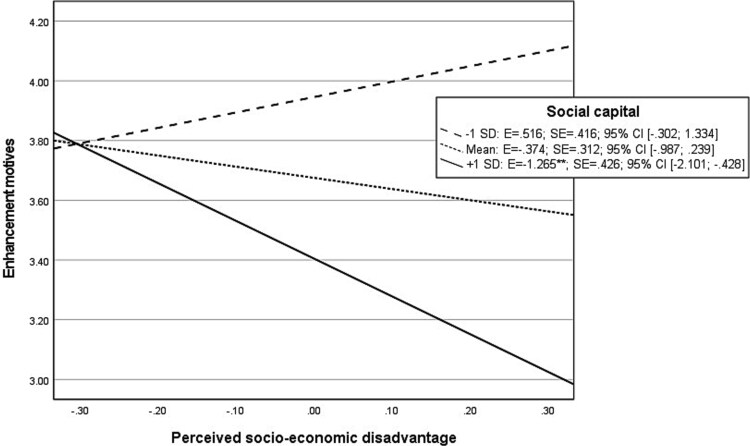
Probed interaction effect perceived socio-economic disadvantage with social capital on enhancement motives in moderated mediation analysis. Footnote: ^*^P < .05; ^**^P < .01; ^***^P < .001.

**Graph 4:**
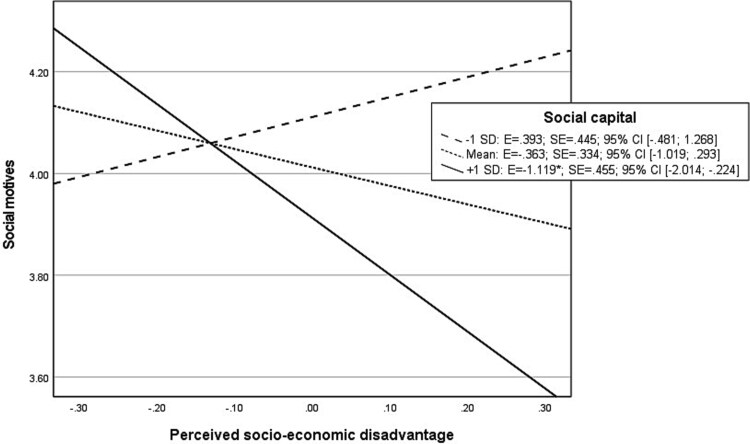
Probed interaction effect of perceived socio-economic disadvantage with social capital on social motives in moderated mediation analysis. Footnote: ^*^P < .05; ^**^P < .01; ^***^P < .001.

**Graph 5:**
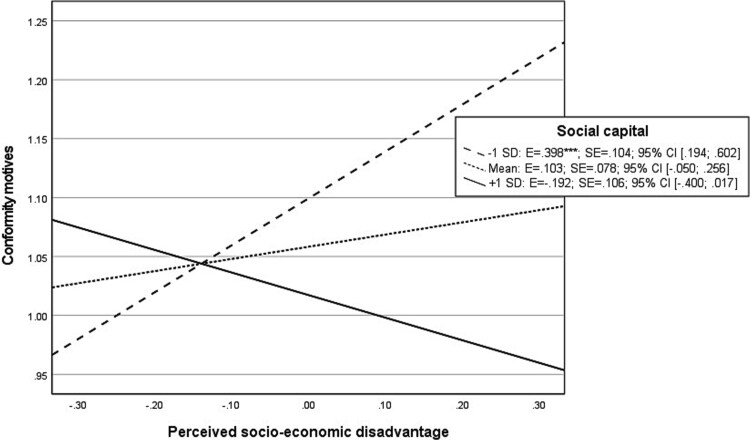
Probed interaction effect of perceived socio-economic disadvantage with social capital on natural log-transformed conformity motives in moderated mediation analysis. Footnote: ^*^P < .05; ^**^P < .01; ^***^P < .001.

The probed interaction effects between socio-economic disadvantage and social capital on enhancement motives display that at high levels of social capital, perceived socio-economic disadvantage showed a significant “negative” association with enhancement (Graph 3). On the other hand, at mean or low levels of social capital, there was no significant association between socio-economic disadvantages and enhancement motives. The same pattern of results was observed for the interaction effect between social capital and socio-economic disadvantage on social motives (see Graph 4). Although a significant interaction between socio-economic disadvantage and social capital on conformity motives was found, indicating a stronger association between socio-economic disadvantage and endorsement of conformity motives when social capital is low (Graph 5), no significant indirect effects were found for conformity motives in explaining the moderation effect between socio-economic disadvantage and social capital on AUD symptoms (Table 3).

Cluster-adjusted sensitivity analysis, testing the pathways of the main model as well as analysis excluding participants below 18 years, did not show differences in results (see https://osf.io/2a8pt/?view_only=ff8808e5182347ba916202792319f3f7).

## Discussion

Adolescents and young adults have a particularly high risk of negative alcohol-related consequences, especially those in a socio-economically disadvantaged position (e.g. Loring 2014, Hua et al. 2020, Probst et al. 2020). To decrease socio-economic inequalities in AUD symptoms, it is therefore important to gain insight into possible factors that may protect against or aggravate AUD symptoms. The current study aimed to gain more insight into the possible moderation effect of social capital in the association between socio-economic disadvantage and AUD symptoms and the underlying psychosocial mechanisms via drinking motives in a sample aged 17–30 years.

Although bivariate correlations showed no associations between socio-economic disadvantage and AUD symptoms, the moderation model results showed that, in our sample, socio-economic disadvantage has different associations with AUD symptoms depending on the level of social capital. In line with the buffering hypothesis, whenever social capital was high, participants reporting higher socio-economic disadvantage showed significantly lower AUD symptoms, whereas this buffering effect of social capital was not found for their more advantaged counterparts. This is in line with results from previous studies that have shown similar buffering effects of social capital on inequalities in other health and well-being outcomes (Uphoff et al. 2013). Our results additionally indicate that among individuals reporting low levels of social capital (compared to high levels), socio-economic disadvantage showed a stronger positive association with AUD symptoms, confirming the cumulative disadvantage hypothesis. Thus, a low social capital puts people in socio-economic disadvantaged positions at increased risk for higher AUD symptoms, which could widen socio-economic inequalities in AUD symptoms.

The moderated mediation results indicated that the mitigating effect of high levels of social capital for individuals in a socio-economic disadvantaged position is associated with their lower endorsement of coping, enhancement, and social motives. Thus, high levels of social capital may protect against using alcohol as a positive (drinking to enhance positive moods) or negative (drinking to decrease negative moods) reinforcement coping mechanism, and it may lead to more opportunities for socializing in ways that do not involve alcohol for people experiencing high socio-economic disadvantage. This non-involvement of alcohol in socializing for people who experience high socio-economic disadvantage and high social capital, may be associated with different normative guidelines when it comes to alcohol use in social situations or with a low affordability of alcohol for this group.

The higher risk for increased AUD symptoms among individuals reporting higher socio-economic disadvantage than their more advantaged counterparts when social capital is low, was associated with their higher endorsement of coping drinking motives only. These results are in line with previous studies showing evidence for higher rates of coping drinking (negative reinforcement; drinking to forget problems) among people in socio-economic disadvantaged positions (Karriker-Jaffe et al. 2016, Martin et al. 2019, Schelleman-Offermans et al. 2022). However, our results extent current knowledge providing preliminary evidence that using alcohol as a coping mechanism to deal with socio-economic disadvantage is dependent on social capital and only occurs when social capital is low. Moreover, only coping drinking motives, not other drinking motives, are used to deal with cumulative disadvantages (low social capital and socio-economic disadvantage).

### Limitations, suggestions for future research, and practical implications

This study has some limitations. First, we used a convenience sample that filled out the questionnaire anonymously. This has limited our data availability regarding more objective aggregate neighborhood factors (e.g. alcohol outlet density) and may have resulted in overestimating current results by not being able to control for possible important covariates. It also may have resulted in specific selection biases (e.g. relatively more respondents from higher socio-economic backgrounds possibly leading to weaker associations between the IV and DV). Secondly, no definite conclusions can be drawn regarding causality since we used cross-sectional data. However, assuming that social determinants of health (socio-economic disadvantage) precede specific psychosocial constructs (drinking motives) that, in turn, precede specific behaviors or symptoms thereof is based on established theory (Cooper 1994, Cox and Klinger 2004, Uphoff et al. 2013) and underlined by findings from previous studies. For instance, cross-lagged panel studies showed that the predictive power of drinking motives for changes in hazardous drinking is much stronger than vice versa (Schelleman-Offermans et al. 2011, Crutzen et al. 2012). Third, we used participants’ perceptions of social capital to predict their own outcome in terms of AUD symptoms. Results may therefore have suffered from same-source bias. Lastly, the current study only included cognitive dimensions of social capital and no other dimensions (e.g. bridging or linking social capital). Future studies should therefore use longitudinal designs and use a broader definition of social capital to better inform interventions.

Results of this study indicate that to decrease socio-economic inequalities in AUD symptoms and related drinking, intervention efforts should target to increase social capital specifically for adolescents and young adults in socio-economic disadvantaged positions. Ways to increase social capital are therefore a promising avenue to explore in future studies. For instance, looking into ways to invest in positive relationships and dialogs between people perceiving a high socio-economic disadvantage to increase trust and cooperation and stimulating cohesive social networks (Villalonga-Olives et al. 2018). One example that has shown potential is a program developed by Andersen et al. (2015) to build social capital at work by promoting stronger connections and trust through 10 weeks of group-based physical exercise during working hours.

## Conclusion

Our results underline the buffering hypothesis and show potential of high levels of social capital to serve as a buffer for socio-economic inequalities in AUD symptoms. Social capital may protect individuals reporting higher socio-economic disadvantage from using alcohol as a positive and negative reinforcement coping mechanism by reducing the endorsement of enhancement and coping motives, respectively. Social capital may also protect against using alcohol as a social lubricant for people experiencing high disadvantage. Additionally, in line with the cumulative disadvantage hypothesis, low levels of social capital showed to increase the socio-economic gradient in AUD symptoms via the increasing use of alcohol as a negative reinforcement coping mechanism.

## Data Availability

The data, syntax file, and outcomes of sensitivity analyses from this study can be accessed via the Open Science Framework at https://osf.io/2a8pt/?view_only=ff8808e5182347ba916202792319f3f7.
